# Analysis of Physeal Fractures from the United States National Trauma Data Bank

**DOI:** 10.3390/children9060914

**Published:** 2022-06-18

**Authors:** Joseph R. Fuchs, Romie F. Gibly, Christopher B. Erickson, Stacey M. Thomas, Nancy Hadley Miller, Karin A. Payne

**Affiliations:** 1 Department of Orthopedics, University of Colorado Anschutz Medical Campus, Aurora, CO 80045, USA; joseph.fuchs@cuanschutz.edu (J.R.F.); rgibly@northwestern.edu (R.F.G.); christopher.erickson@cuanschutz.edu (C.B.E.); stacey.m.thomas@cuanschutz.edu (S.M.T.); nancy.miller@childrenscolorado.org (N.H.M.); 2 McGaw Medical Center, Northwestern University, Chicago, IL 60611, USA; 3 Division of Orthopaedic Surgery and Sports Medicine, Ann & Robert H. Lurie Children’s Hospital of Chicago, Chicago, IL 60611, USA; 4 Department of Bioengineering, University of Colorado Anschutz Medical Campus, Aurora, CO 80045, USA; 5 Musculoskeletal Research Center, Children’s Hospital Colorado, Aurora, CO 80045, USA; 6 Gates Center for Regenerative Medicine, University of Colorado Anschutz Medical Campus, Aurora, CO 80045, USA

**Keywords:** physeal, physis, fracture, trauma, long-bone fractures in children

## Abstract

Background: Pediatric long-bone physeal fractures can lead to growth deformities. Previous studies have reported that physeal fractures make up 18–30% of total fractures. This study aimed to characterize physeal fractures with respect to sex, age, anatomic location, and Salter–Harris (SH) classification from a current multicenter national database. Methods: A retrospective cohort study was performed using the 2016 United States National Trauma Data Bank (NTDB). Patients ≤ 18 years of age with a fracture of the humerus, radius, ulna, femur, tibia, or fibula were included. Results: The NTDB captured 132,018 patients and 58,015 total fractures. Physeal fractures made up 5.7% (3291) of all long-bone fractures, with males accounting for 71.0% (2338). Lower extremity physeal injuries comprised 58.6% (1929) of all physeal fractures. The most common site of physeal injury was the tibia comprising 31.8% (1047), 73.9% (774) of which were distal tibia fractures. Physeal fractures were greatest at 11 years of age for females and 14 years of age for males. Most fractures were SH Type II fractures. Discussion and Conclusions: Our analysis indicates that 5.7% of pediatric long-bone fractures involved the physis, with the distal tibia being the most common. These findings suggest a lower incidence of physeal fractures than previous studies and warrant further investigation.

## 1. Introduction

It is estimated that 18% to 30% of all pediatric fractures involve the physis, a cartilaginous area at the ends of long bones [1,2]. Physeal fractures are of particular concern as they can lead to partial or complete physeal arrest, resulting in angular deformities or limb-length discrepancy. Understanding the epidemiology of physeal injuries is important for the early identification of these injuries, as late presentation can result in complex deformities that can lead to greater challenges in achieving good clinical results [3,4].

**Figure 1:**
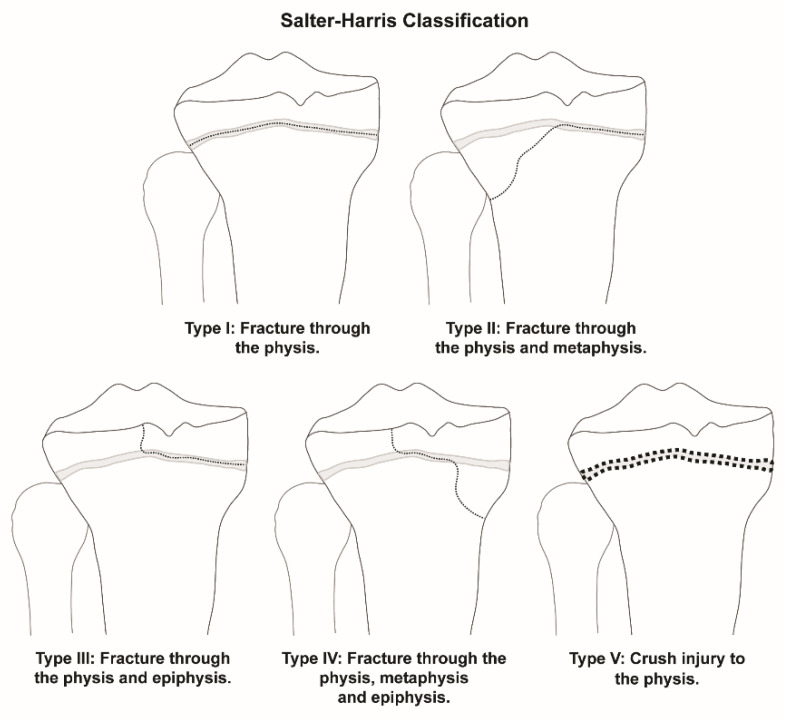
Salter–Harris Classification of pediatric physeal fractures. The five fracture types are shown with the fracture line represented by a dotted line.

Previous epidemiology studies looking at sex differences have indicated that a predominance of physeal injuries occur in males [1,2,3]. The distribution of anatomic locations of physeal fractures for long bones of the appendicular skeleton has been explored as well, with most fractures occurring in the upper extremities, especially of the radius [1,3,5]. This observation has led to outcomes research analyzing the treatment of these fractures, such as outcomes based on body habitus [6]. Fractures that involve the physis are classified according to the Salter–Harris (SH) classification system, which grades fractures according to the involvement of the physis, metaphysis, and epiphysis (Figure 1). Past studies have shown that SH Type II fractures are the most common type of physeal injury [1,2,3].

Additional studies have analyzed trends in physeal fractures such as age and sex predominance for specific long bones and anatomic locations [7,8,9,10,11,12]. Few, however, have compared all physeal fractures and identified which anatomic locations are at a higher risk for injury [1,2,3]. Comparison of all long-bone fractures to physeal fractures according to age and sex has also rarely been studied [1]. While most of these studies have focused on populations from a single institution, a larger-scale study was conducted in Olmstead County, MN, between 1979–1988 [3]. This study indicated that physeal injuries peak between the ages of 12–14 years. Since that time, motor vehicle technology and sports intensity have evolved, potentially altering the prevalence and severity of physeal injuries [13]. This has been explored in previous work that has analyzed the epidemiology of pediatric fractures based on age, type, and anatomic location at a single institution over time [14]. However, analyzing a more recent and larger dataset from multiple institutions could provide a better representation of the current epidemiology of physeal fractures.

The aim of this study was to analyze trends of pediatric long-bone fractures from a current United States multicenter national database to better characterize both physeal and nonphyseal fractures with respect to sex, age, anatomic location, and SH classification.

## 2. Materials and Methods

Following approval from the Institutional Review Board (COMIRB 20-0020), a retrospective cohort study was performed using the American College of Surgeons (ACS) United States National Trauma Data Bank (NTDB). The NTDB consists of patient demographics, ICD-10 codes for all injuries presenting within 14 days of occurrence, trauma center designation, and population treated [15]. The NTDB is the largest aggregation of U.S. trauma registry data and was designed to establish a national standard for trauma data [16]. The NTDB has been utilized to study the epidemiology of adult patients with hip fractures and spinal injury in pediatric populations [17,18]. The ACS utilizes the National Trauma Data Standard and validates data annually to ensure incomplete and nonsensical data are not included [19].

**Figure 2:**
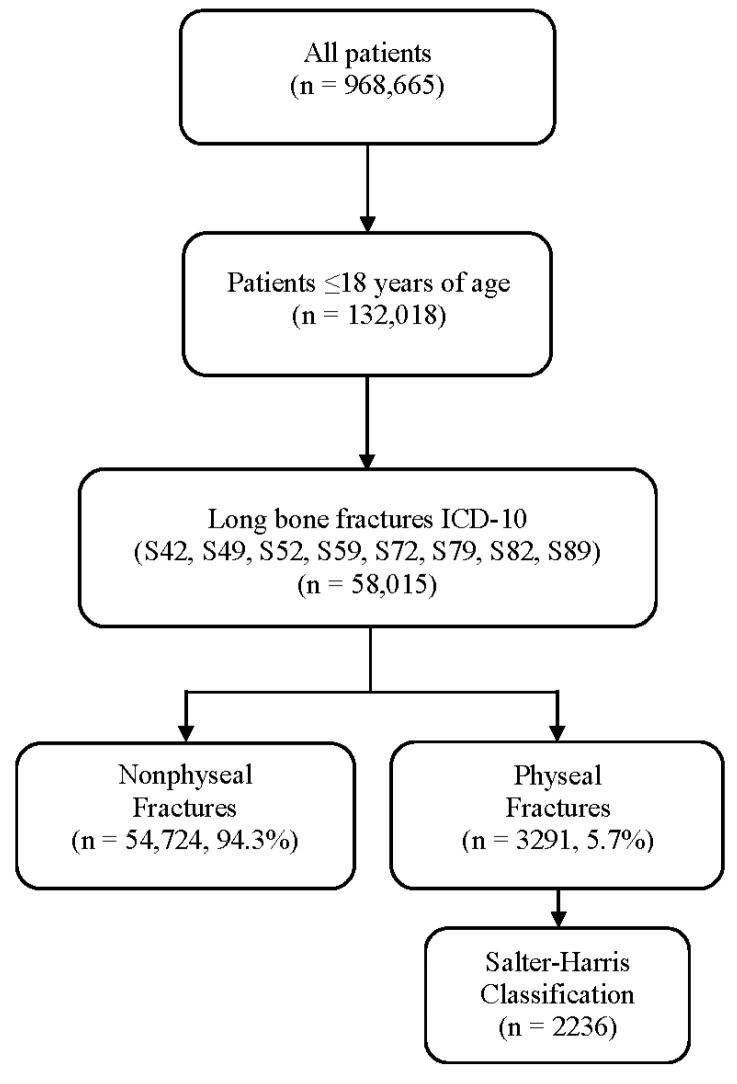
Flowchart of inclusion criteria using data from the NTDB.

The data, volunteered from over 740 institutions in the United States and Puerto Rico, are comprised of approximately two hundred Level I, II, III or IV trauma centers each and 36 Level I or II pediatric-only centers. Using the 2016 version of the NTDB, data given in CSV files were loaded into Microsoft Access. In the NTDB each patient is coded with a unique inclusion key and ICD-10 diagnosis code, along with demographic data. ICD-10 diagnosis codes based on the clinical presentation and incorporating clinical data are coded by the trauma registrar or data abstractor at each institution. This allowed patients ≤ 18 years to be identified. From those, records with ICD-10 diagnosis codes for long-bone fractures of the humerus, radius, ulna, femur, tibia, or fibula (S42, S49, S52, S59, S72, S79, S82, S89) were selected (Figure 2). Within the ICD-10 diagnosis codes for long-bone fractures are more specific codes for physeal injuries and SH Type classification [20]. These were utilized to categorize long-bone fractures more specifically into nonphyseal fractures or physeal fractures (excluding slipped capital femoral epiphysis). All physeal ICD-10 codes for each long bone were included. Physeal fractures were further categorized using the SH classification, when available (Figure 2). The detailed ICD-10 codes also allowed for an anatomic location such as the proximal and distal end of bones to be differentiated. For example, S49.0 codes for “physeal fracture of upper end of humerus” and S49.1 codes for “physeal fracture of lower end of humerus”. The range of ICD-10 diagnosis codes analyzed ensured that all long-bone fractures were included in the study, with further granularity for anatomic location and physeal involvement based on more specific coding. Once the dataset was generated, data were analyzed based on sex, age, anatomic location, and fracture type for long-bone fractures. Age groups (0–4, 5–8, 9–12, and 13–18 years) were utilized to organize the data. These groups were determined based on groups utilized in previous studies and to highlight common ages for physeal injuries as described in the literature [2,3]. 

## 3. Results

The 2016 version of the NTDB consisted of entries for 968,665 patients. Of those, 132,018 patients were identified to be ≤18 years. 42,429 of those patients had at least one long-bone fracture code associated with their entry, resulting in a total of 58,015 fractures (Figure 2).

When analyzing all long-bone fractures, regardless of physeal involvement, males accounted for 65.6% (38,053) of long bone fractures while females comprised 34.4% (19,962). The total long-bone fracture data were then categorized into nonphyseal or physeal fractures. Nonphyseal fractures comprised 94.3% (54,724) of the long-bone fractures included in this study, and males accounted for 65.3% (35,715) of all nonphyseal fractures. Fractures involving the physis made up 5.7% (3291) of the total fractures reported in this study (6.1% in males and 4.8% in females). Males accounted for 71.0% (2338) of all physeal fractures. While SH classification information was not available for all fractures, 67.9% (2236) of the overall physeal fractures were associated with an SH class by ICD-10 coding (Figure 2).

**Figure 3:**
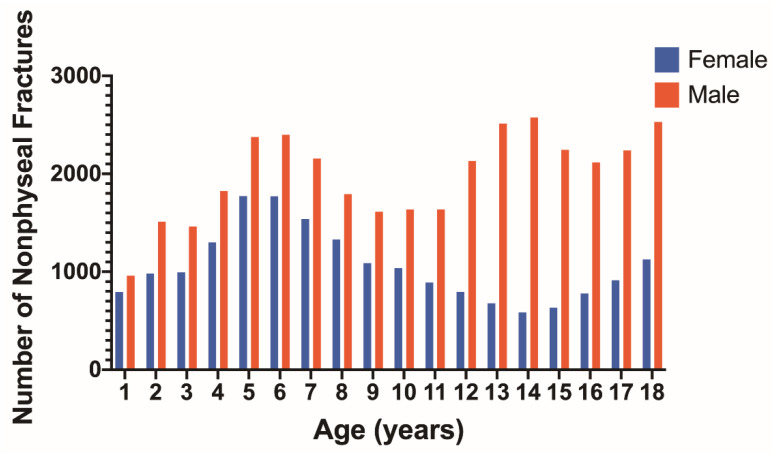
Number of nonphyseal fractures at each age for females (blue) and males (red).

Nonphyseal fractures for each age by sex show a relative peak at the ages of 5–6 years for both males and females (Figure 3). Another peak appears for males at 13–14 years of age which is not seen for females. The distribution of nonphyseal fractures according to long bone and separated by age is represented for males in Table 1 and females in Table 2. Upper extremity nonphyseal injuries accounted for 56.6% (20,206) of all nonphyseal fractures in males and 65.6% (12,476) in females. Nonphyseal fractures occurred most frequently in the humerus, followed by the femur, radius, ulna, and tibia which all had similar occurrences. Nonphyseal fractures to the fibula occurred less frequently. 

**Figure 4:**
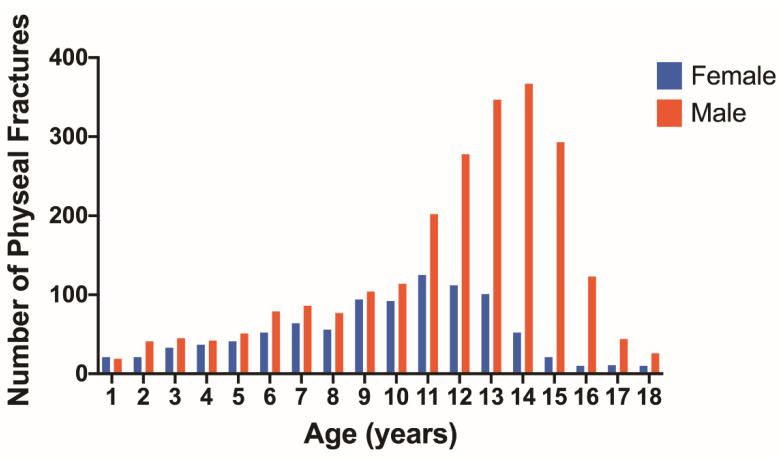
Number of physeal fractures at each age for females (blue) and males (red).

The number of physeal fractures at each age for males and females is shown in Figure 4. A peak occurs around the age of 11 for females and at the age of 14 for males. The number of physeal fractures is much higher for males than females after 10 years of age. The number and percentage of physeal fractures according to age, long bone, and proximal or distal location is shown for males in Table 3 and females in Table 4. In males, the largest percentage of physeal fractures were observed in the distal tibia and distal radius in the 13–18 age group (Table 3). Females also had the greatest number of physeal fractures in these locations, but they mostly occurred in the 9–12 age group (Table 4). 

**Figure 5:**
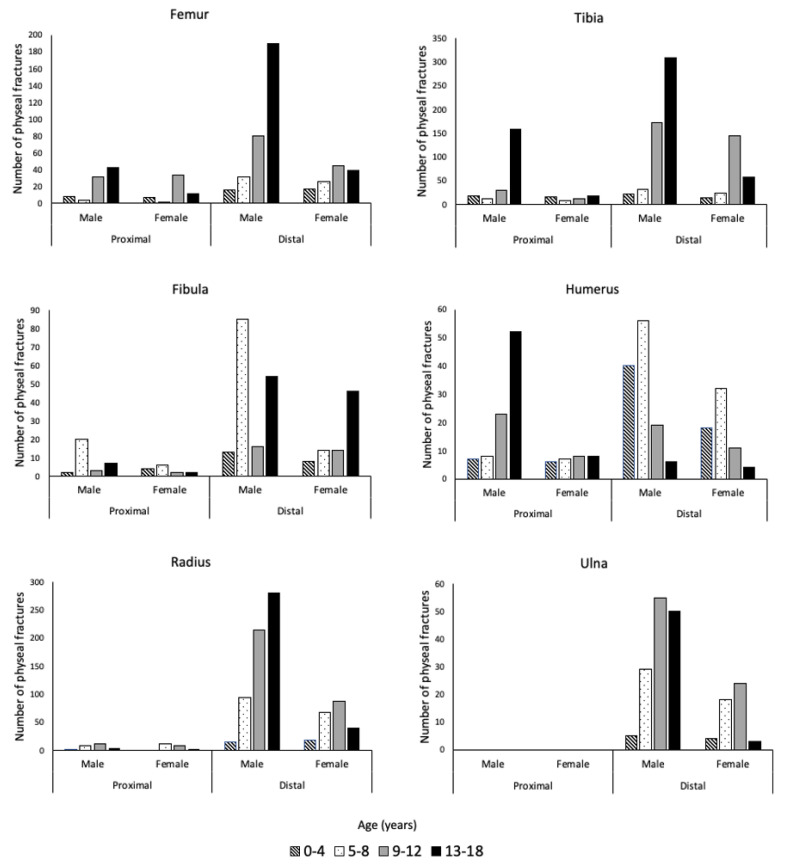
The number of physeal fractures for the femur, tibia, fibula, humerus, radius, and ulna, separated by sex, anatomic location (proximal and distal), and age group.

The breakdown of all physeal fractures by bone and further separated by proximal or distal location, sex, and age can be seen in Figure 5. Lower extremity fractures comprised 58.6% (1929/3291) of physeal injuries. The most common long bone with physeal injury was the tibia, accounting for 31.8% (1047/3291) of all physeal fractures. Of these fractures, the distal tibia was predominantly injured, accounting for 71.0% (534/752) and 81.4% (240/295) of tibial physeal fractures for males and females, respectively. In females, tibial physeal fractures occurred most often between the ages of 9–12 years, while for males, 13–18 years made up the largest share. The second most common site of physeal fracture for both males and females was the radius. Similar to the tibia, the distal end of the bone was more highly affected than the proximal end. A physeal fracture affecting the distal radius occurred in 95.7% (605/632) and 90.3% (214/237) of total physeal fractures of the radius for males and females, respectively. Physeal fractures of the radius occurred most frequently for females from the ages of 9–12 and for males from ages 13–18.

The SH classification was available for 2236 physeal fractures and analysis of these data indicated that SH class II fractures were the most common physeal fractures, comprising 69.0% (1543) of fractures (Table 5). SH class I, III, and IV each made up less than 12% of physeal fractures.

## 4. Discussion

Previous epidemiology studies of physeal fractures have largely been completed through an analysis of fractures at single academic institutions [1,2,3,21]. While this provides useful information, a more comprehensive view of physeal fractures can be obtained using data from the NTDB. The NTDB data come from 747 institutions, comprising 968,665 patient records from all regions of the United States. Therefore, this study is derived from a much larger and more varied sample of patients which allows for a better understanding of physeal fractures.

Males accounted for a majority of all fractures with peak nonphyseal fracture occurrence at 5–6 years and 13–14 years, which is similar to previous studies [21,22]. Studies have found that males generally account for 55–65% of all fractures, which is similar to our results [1,22]. In the case of nonphyseal fractures affecting females, previous studies have noted a peak at 11 years compared to the results of this study which demonstrated a peak at 5 and 6 years [2,21]. This inconsistency is possibly due to differences such as sports participation based on age and sex across times and in various countries [2,21].

In this study, the most affected long bone for nonphyseal fractures is the humerus. This deviates from studies that have found the radius to comprise 30–60% of nonphyseal fractures [1,2]. This is a unique finding that warrants further investigation to clearly establish this predominance and may relate to the subset of patients presenting to centers participating in the NTDB, with a potential for higher acuity of trauma.

Our analysis indicated that physeal fractures made up 5.7% of the total fractures, which is less than the rate of 18–30% found in previous studies [1,2,3,23]. One reason for this discrepancy could be that the sample was derived from long-bone fractures, while many previous studies included fractures of the phalanges. However, in Mann et al. the rate was 30% based solely on long-bone data [1]. Another factor that should be considered is the ages included in each study. For example, Peterson et al. utilized a sample of patients 0–21 years while Worlock et al. included patients 0–12 years [3,24]. However, when altering the age range analyzed using the NTDB data to include age ranges from 0–14 years, 0–15 years, 0–16 years, or 0–17 years, the maximum percentage of physeal fractures found is less than 7%. The NTDB is comprised of hundreds of institutions and thousands of patients which can be contrasted with most studies that were conducted at single institutions that are largely academic centers. It is possible that previous studies with smaller sample sizes may have seen higher rates of complicated injuries. It is also possible that not all physeal fractures would present at a trauma center. Therefore, the rate of physeal fractures compared to overall fractures may be lower than previously thought but further investigation is needed.

The most common age for physeal fractures was 14 years for males and 11 years for females. This has been noted in previous studies and has been attributed to the weakness of the growth plate during puberty [2]. Of physeal fractures, males accounted for 71.0%. This is consistent with studies that demonstrate a male predominance of 66–75% [1,3,23]. Lower extremity physeal injuries comprised a majority of injuries for both males and females. This is consistent with a previous study of long-bone physeal fractures which demonstrated that 54.78% of fractures were in the lower extremity [1]. In this study, the tibia was the long bone with the greatest percentage of physeal fractures, accounting for greater than 30% of injuries. The second most common long bone with physeal fractures was the radius with 26.4%. This deviates from previous studies, which found a predominance of physeal fractures of the radius, comprising 28–30% of long-bone physeal fractures [1,3]. This difference may be due to the NTBD being comprised of data from trauma centers that see higher acuity injuries compared to primary care settings, as tibial fractures result in difficulty with ambulation.

Tibial physeal fractures were more commonly fractured at the distal end than the proximal end. This is consistent with previous studies with distal injuries accounting for a majority (96%) of tibial physeal fractures [1]. This was also seen for the radius, with distal physeal fractures comprising greater than 90% of physeal injuries, consistent with previous estimates of 86.36% [1]. Therefore, distal tibial and radial injuries should be closely followed to ensure the physis is not impacted. A recent study of SH Type II fractures of the distal tibia suggested that fracture displacement greater than 3 mm should be treated with closed reduction and casted [25]. If displacement remains greater than 3 mm, open reduction should be performed. The study found a small risk of growth arrest after closed reduction for displaced fractures. Thus, initial recognition, treatment, and follow-up of distal tibial and radial physeal fractures by a pediatric orthopedist can help avoid long-term complications such as angular deformity and limb-length discrepancy in pediatric populations.

SH Type II fractures were the dominant type of physeal injuries observed in this study, which has been a consistent pattern throughout the decades [1,2,3,23]. SH Types I, III, and IV each accounted for 12% or less of physeal fractures, which has been noted previously [1].

Previous studies have looked at the incidence of physeal fractures through direct retrospective analysis of radiographs [1,2,3,23]. The NTDB for 2016 is the first year that contains ICD-10 codes, which have many subclassifications for physeal injuries. Previously utilized ICD-9 codes did not include specifications for proximal or distal physis, an essential piece of knowledge when addressing physeal fractures of long bones. Through proper ICD-10 coding based on radiographic reads, it is possible to have a better understanding of physeal injuries that is standardized across institutions. This is beneficial for future studies as researchers can more quickly sort through data without having to analyze numerous radiographs that will not meet study inclusion criteria. A limitation of this information, however, is that the ICD-10 diagnosis codes utilized in this analysis are reliant on radiologic reads and radiographic imaging could not be independently verified. Therefore, errors in the assignment of ICD-10 codes during data entry may have impacted our results. However, the ACS validates the NTDB by not including missing or nonsensical data. Future studies should compare ICD-10 diagnosis codes to independently verified radiographic reads to determine if differences exist.

Despite the ability of the NTDB to provide an estimate for physeal injuries across hundreds of institutions within the country, there are some limitations to the data. The most important limitation is that the data are voluntarily provided by trauma centers. Therefore, there are differences in the number of patients seen at each institution, as well as the number of institutions for each region. Thus, this study does not provide a national representation of injuries. These differences do not allow for a denominator to be determined which would provide estimates of the incidence of pediatric physeal injuries nationally. However, this is a similar limitation to previous studies that describe the occurrence of physeal injuries at singular institutions.

## 5. Conclusions

In conclusion, we found that 5.7% of all pediatric long-bone fractures involved the physis, with the distal tibia the most common site of injury. Utilizing this knowledge physicians should be vigilant about these injuries to prevent future complications commonly seen in physeal fractures such as bony bar formation and limb-length discrepancy.

## Figures and Tables

**Table 1:** Nonphyseal fractures for males according to age and long bone. N = 35,715.

Age	Femur n (%)	Tibia n (%)	Fibula n (%)	Humerus n (%)	Radius n (%)	Ulna n (%)
0–4	2077 (5.8)	417 (1.2)	166 (0.5)	2190 (6.1)	461 (1.3)	450 (1.3)
5–8	987 (2.8)	485 (1.4)	296 (0.8)	3820 (10.7)	1610 (4.5)	1524 (4.3)
9–12	1012 (2.8)	873 (2.4)	616 (1.7)	1089 (3.0)	1811 (5.1)	1618 (4.5)
13–18	2657 (7.4)	3624 (10.1)	2299 (6.4)	1284 (3.6)	2290 (6.4)	2059 (5.8)
**Total**	6733 (18.9)	5399 (15.1)	3377 (9.5)	8383 (23.5)	6172 (17.3)	5651 (15.8)

**Table 2:** Nonphyseal fractures for females according to age and long bone. N = 19,009.

Age	Femur n (%)	Tibia n (%)	Fibula n (%)	Humerus n (%)	Radius n (%)	Ulna n (%)
0–4	858 (4.5)	341 (1.8)	127 (0.7)	2076 (10.9)	315 (1.7)	352 (1.9)
5–8	497 (2.6)	353 (1.9)	206 (1.1)	3308 (17.4)	1059 (5.6)	990 (5.2)
9–12	422 (2.2)	501 (2.6)	313 (1.6)	888 (4.7)	903 (4.8)	784 (4.1)
13–18	999 (5.3)	1155 (6.1)	761 (4.0)	553 (2.9)	652 (3.4)	596 (3.1)
**Total**	2776 (14.6)	2350 (12.4)	1407 (7.4)	6825 (35.9)	2929 (15.4)	2722 (14.3)

**Table 3:** Physeal fractures for males according to age, long bone, and location (proximal or distal). N = 2338.

Bone n (%)												
	Femur		Tibia		Fibula		Humerus		Radius		Ulna	
Age	P	D	P	D	P	D	P	D	P	D	P	D
0–4	8 (0.3)	16 (0.7)	17 (0.7)	22 (0.9)	2 (0.1)	13 (0.6)	7 (0.3)	40 (1.7)	2 (0.1)	15 (0.6)	0 (0.0)	5 (0.2)
5–8	4 (0.2)	31 (1.3)	13 (0.6)	31 (1.3)	20 (0.9)	85 (3.6)	8 (0.3)	56 (2.4)	8 (0.3)	94 (4.0)	0 (0.0)	29 (1.2)
9–12	32 (1.4)	80 (3.4)	29 (1.2)	173 (7.4)	3 (0.1)	16 (0.7)	23 (1.0)	19 (0.8)	12 (0.5)	214 (9.2)	0 (0.0)	55 (2.4)
13–18	43 (1.8)	190 (8.1)	159 (6.8)	308 (13.2)	7 (0.3)	54 (2.3)	52 (2.2)	6 (0.3)	5 (0.2)	282 (12.1)	0 (0.0)	50 (2.1)

P = proximal, D = distal.

**Table 4:** Physeal fractures for females according to age, long bone, and location (proximal or distal). N = 953.

Bone n (%)												
	Femur		Tibia		Fibula		Humerus		Radius		Ulna	
Age	P	D	P	D	P	D	P	D	P	D	P	D
0–4	7 (0.7)	17 (1.8)	16 (1.7)	14 (1.5)	4 (0.4)	8 (0.8)	6 (0.6)	18 (1.9)	0 (0.0)	18 (1.9)	0 (0.0)	4 (0.4)
5–8	2 (0.2)	26 (2.7)	8 (0.8)	24 (2.5)	6 (0.6)	14 (1.5)	7 (0.7)	32 (3.4)	12 (1.3)	68 (7.1)	0 (0.0)	18 (1.9)
9–12	34 (3.6)	45 (4.7)	13 (1.4)	144 (15.1)	2 (0.2)	14 (1.5)	8 (0.8)	11 (1.2)	8 (0.8)	88 (9.2)	0 (0.0)	24 (2.5)
13–18	12 (1.3)	39 (4.1)	18 (1.9)	58 (6.1)	2 (0.2)	46 (4.8)	8 (0.8)	4 (0.4)	3 (0.3)	40 (4.2)	0 (0.0)	3 (0.3)

P = proximal, D = distal.

**Table 5:** Salter–Harris classification of physeal injuries based on long bone. N = 2236.

	SH I (%)	SH II (%)	SH III (%)	SH IV (%)
**Femur**	96 (4.3)	231 (10.3)	31 (1.4)	16 (0.7)
**Tibia**	36 (1.6)	448 (20)	119 (5.3)	204 (9.1)
**Fibula**	39 (1.7)	120 (5.4)	0 (0)	0 (0)
**Humerus**	32 (1.4)	119 (5.3)	0 (0)	20 (0.9)
**Radius**	50 (2.2)	546 (24.4)	13 (0.6)	16 (0.7)
**Ulna**	9 (0.4)	79 (3.5)	8 (0.4)	4 (0.2)
**Total**	262 (11.7)	1543 (69.0)	171 (7.6)	260 (11.6)

## Data Availability

Data available upon reasonable request.
